# Supplementary material for: End Sequence Analysis Toolkit (ESAT) expands the extractable information from single-cell RNA-seq data
Source: Genome Res. 2016 Oct;26(10):1397–410. doi: 10.1101/gr.207902.116 (PMC5052061; doi:10.1101/gr.207902.116)
Supplement: Supplemental Material [file supp_gr.207902.116_Supp_Materials.tar.gz › ESAT-esat_v0.1/doc/html/classbroad_1_1core_1_1sequence_1_1_transcribed_sequence_composition.html]

ESAT: broad.core.sequence.TranscribedSequenceComposition Class Reference


ESAT


- Main Page
- Related Pages
- Packages
- Classes
- Files

- Class List
- Class Index
- Class Hierarchy
- Class Members

All Classes Namespaces Files Functions Variables Enumerator Pages

- broad
- core
- sequence
- TranscribedSequenceComposition

Classes |
Public Member Functions |
Static Public Member Functions |
Protected Attributes |
List of all members

broad.core.sequence.TranscribedSequenceComposition Class Reference

Collaboration diagram for broad.core.sequence.TranscribedSequenceComposition:

[legend]

|  |  |
| --- | --- |
| Public Member Functions | |
|  | TranscribedSequenceComposition (String bamFile, String geneBedFile, String genomeFasta, TranscriptionRead transcriptionRead) throws IOException |
|  | |
|  | TranscribedSequenceComposition (String bamFile, String geneBedFile, String bedFileRegionsOfInterest, String genomeFasta, TranscriptionRead transcriptionRead) throws IOException |
|  | |
| char | getTranscribedBaseAtBeginningPosition (Gene parent, Alignment read, int offset) throws IOException |
|  | |

|  |  |
| --- | --- |
| Static Public Member Functions | |
| static void | main (String[] args) throws IOException |
|  | |

|  |  |
| --- | --- |
| Protected Attributes | |
| Collection< String > | allNucleotides |
|  | |

## Detailed Description

Author
:   prussell

## Constructor & Destructor Documentation

|  |  |  |  |
| --- | --- | --- | --- |
| broad.core.sequence.TranscribedSequenceComposition.TranscribedSequenceComposition | ( | String | *bamFile*, |
|  |  | String | *geneBedFile*, |
|  |  | String | *genomeFasta*, |
|  |  | TranscriptionRead | *transcriptionRead* |
|  | ) |  | throws IOException |

Parameters
:   |  |  |
    | --- | --- |
    | bamFile | Bam file |
    | geneBedFile | Bed file of genes |
    | genomeFasta | Genome fasta file |
    | transcriptionRead | Transcription read |

Exceptions
:   |  |  |
    | --- | --- |
    | IOException |  |

Here is the caller graph for this function:

|  |  |  |  |
| --- | --- | --- | --- |
| broad.core.sequence.TranscribedSequenceComposition.TranscribedSequenceComposition | ( | String | *bamFile*, |
|  |  | String | *geneBedFile*, |
|  |  | String | *bedFileRegionsOfInterest*, |
|  |  | String | *genomeFasta*, |
|  |  | TranscriptionRead | *transcriptionRead* |
|  | ) |  | throws IOException |

Parameters
:   |  |  |
    | --- | --- |
    | bamFile | Bam file |
    | geneBedFile | Bed file of genes |
    | bedFileRegionsOfInterest | Bed file of regions within genes |
    | genomeFasta | Genome fasta file |
    | transcriptionRead | Transcription read |

Exceptions
:   |  |  |
    | --- | --- |
    | IOException |  |

## Member Function Documentation

|  |  |  |  |
| --- | --- | --- | --- |
| char broad.core.sequence.TranscribedSequenceComposition.getTranscribedBaseAtBeginningPosition | ( | Gene | *parent*, |
|  |  | Alignment | *read*, |
|  |  | int | *offset* |
|  | ) |  | throws IOException |

Get transcribed base of parent gene at first position of alignment

Parameters
:   |  |  |
    | --- | --- |
    | parent | Parent gene |
    | read | Alignment |
    | offset | Offset along the parent transcript from the beginning position |

Returns
:   The transcribed base or N if read orientation does not match parent gene strand

Exceptions
:   |  |  |
    | --- | --- |
    | IOException |  |

|  |  |  |  |  |  |
| --- | --- | --- | --- | --- | --- |
| static void broad.core.sequence.TranscribedSequenceComposition.main | ( | String[] | *args* | ) | throws IOException |

static

Parameters
:   |  |  |
    | --- | --- |
    | args |  |

Exceptions
:   |  |  |
    | --- | --- |
    | IOException |  |

Here is the call graph for this function:

## Member Data Documentation

Collection<String> broad.core.sequence.TranscribedSequenceComposition.allNucleotides

protected

---

The documentation for this class was generated from the following file:

- java/broad/core/sequence/TranscribedSequenceComposition.java


---

Generated on Fri Aug 15 2014 15:10:56 for ESAT by  

 1.8.7
